# Supplementary material for: Dynamic Allocation of Carbon Storage and Nutrient-Dependent Exudation in a Revised Genome-Scale Model of Prochlorococcus
Source: Front Genet. 2021 Feb 9;12:586293. doi: 10.3389/fgene.2021.586293 (PMC7900632; doi:10.3389/fgene.2021.586293)
Supplement: Supplementary Figure 1 — Estimation of kinetic parameters for the uptake of ammonium in Prochlorococcus. (A) All combinations of Km and Vmax along the red trajectory matches the observed gross growth rate of 0.5 d–1 (Grossowicz et al., 2017). However, when we compare the dynamics of cell density (B) and ammonium concentration (C) we find that the best overall prediction is achieved using Km = 0.39 mM and Vmax = 0.9 mmol gDW–1 h–1 (marked by an orange dot in A). [file Presentation_1.pdf]

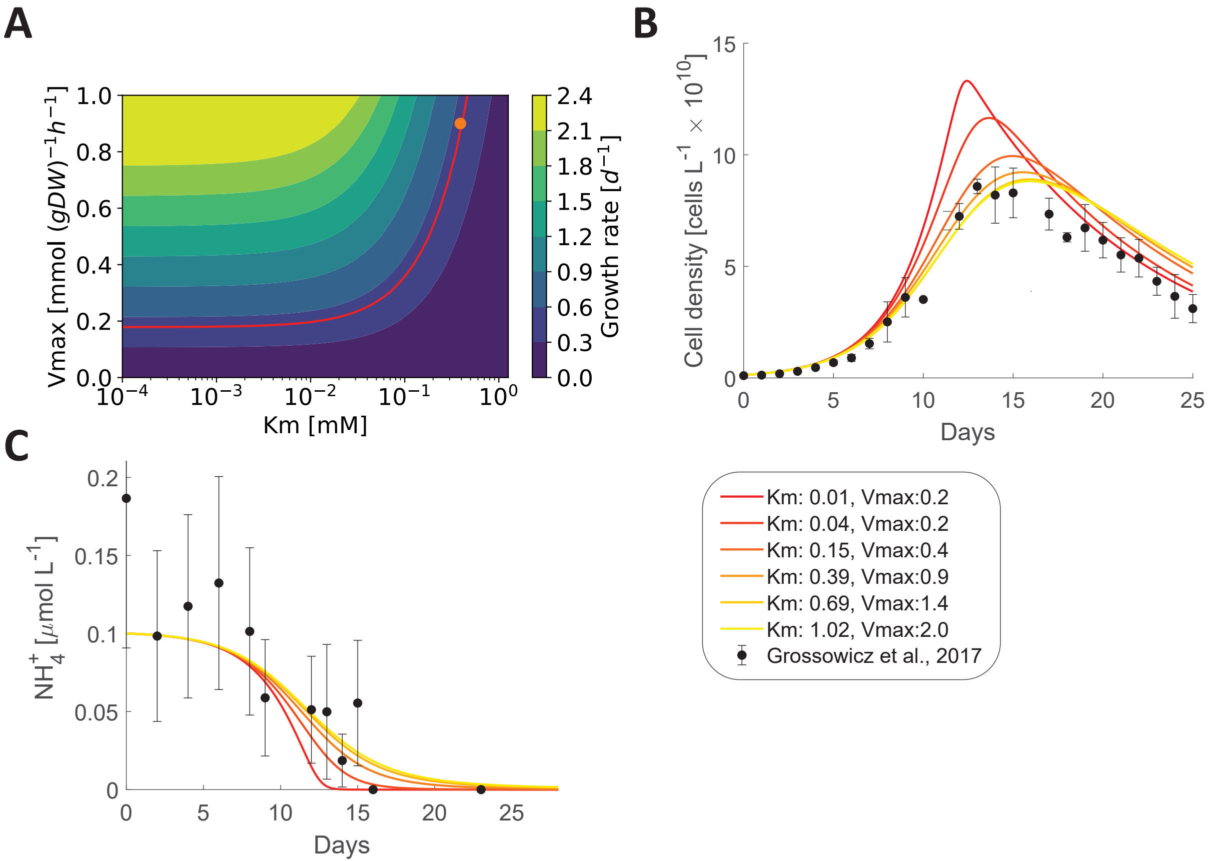
A
B
C
Vmax [mmol (gDW)$^{-1}h^{-1}$]
Km [mM]
Growth rate [d^{-1}]
1.0
0.8
0.6
0.4
0.2
0.0
10^{-4}
10^{-3}
10^{-2}
10^{-1}
10^{0}
2.4
2.1
1.8
1.5
1.2
0.9
0.6
0.3
0.0
Cell density [cells L^{-1} × 10^{10}]
Days
15
10
5
0
0
5
10
15
20
25
NH$_4^+$ [μmol L^{-1}]
0.2
0.15
0.1
0.05
0
Km: 0.01, Vmax:0.2
Km: 0.04, Vmax:0.2
Km: 0.15, Vmax:0.4
Km: 0.39, Vmax:0.9
Km: 0.69, Vmax:1.4
Km: 1.02, Vmax:2.0
Grossowicz et al., 2017

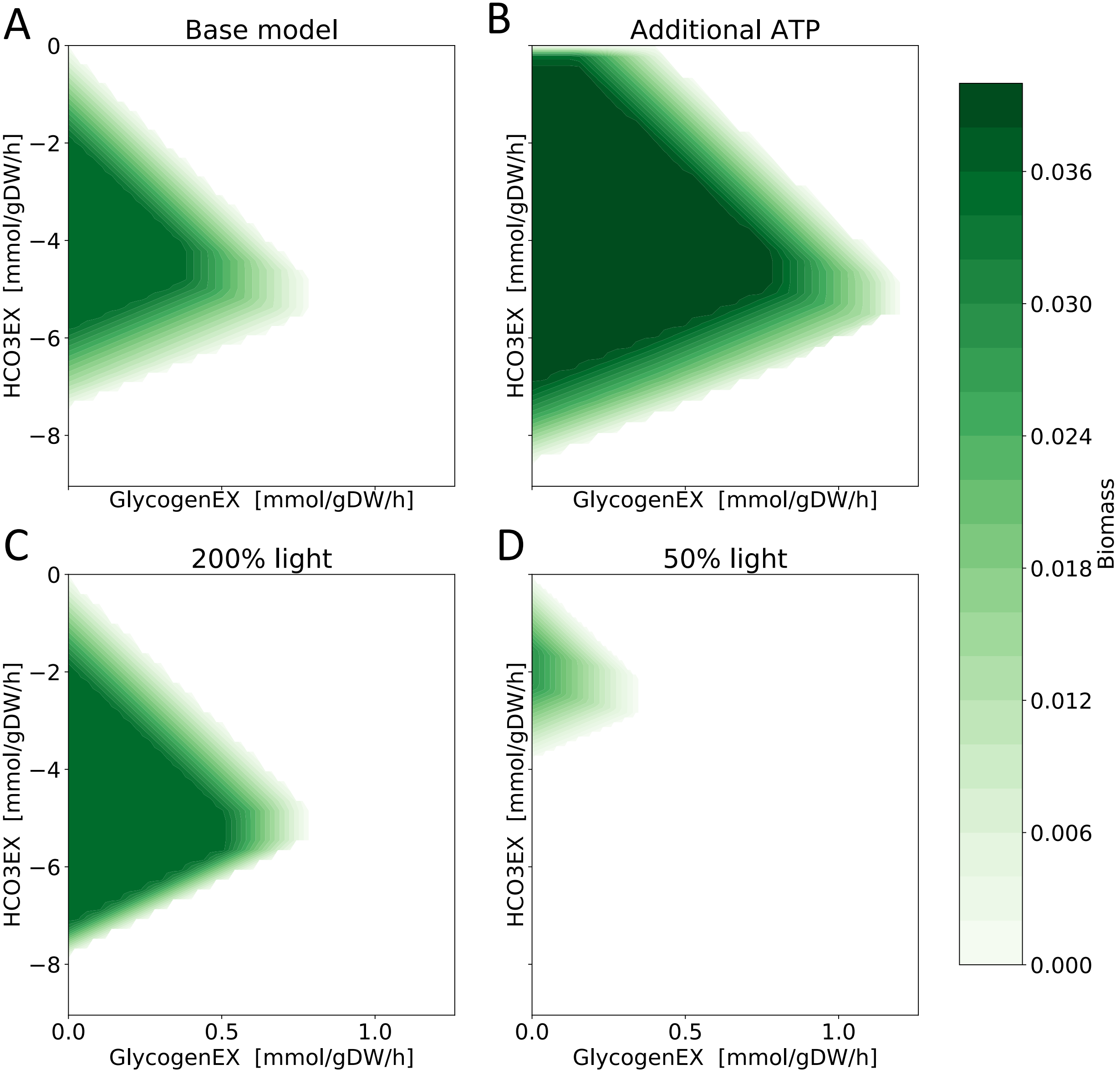
A
Base model
B
Additional ATP
C
200% light
D
50% light
HCO3EX [mmol/gDW/h]
GlycogenEX [mmol/gDW/h]
0
−2
−4
−6
−8
0.0
0.5
1.0
Biomass
0.000
0.006
0.012
0.018
0.024
0.030
0.036

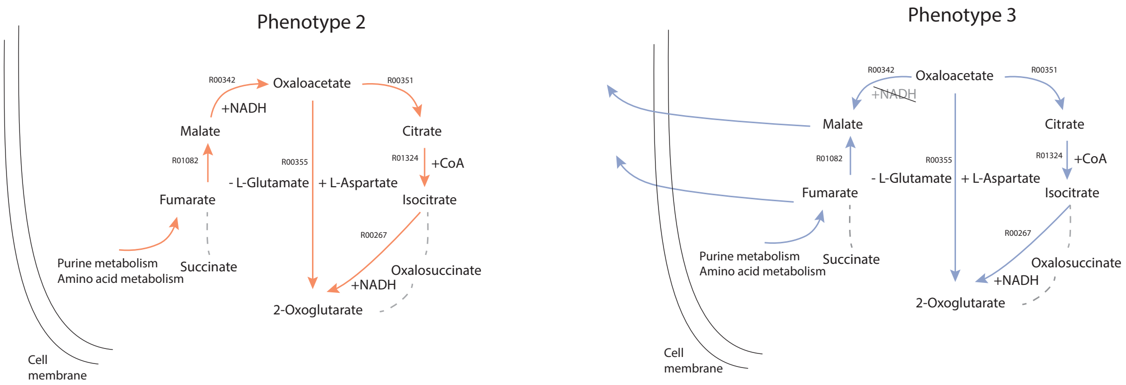
Phenotype 2
R00342
Oxaloacetate
R00351
+NADH
Malate
Citrate
R01082
R00355
R01324
+CoA
- L-Glutamate
+ L-Aspartate
Fumarate
Isocitrate
R00267
Purine metabolism
Amino acid metabolism
Succinate
Oxalosuccinate
+NADH
2-Oxoglutarate
Cell
membrane
Phenotype 3
R00342
Oxaloacetate
R00351
+NADH
Malate
Citrate
R01082
R00355
R01324
+CoA
- L-Glutamate
+ L-Aspartate
Fumarate
Isocitrate
R00267
Purine metabolism
Amino acid metabolism
Succinate
Oxalosuccinate
+NADH
2-Oxoglutarate
Cell
membrane

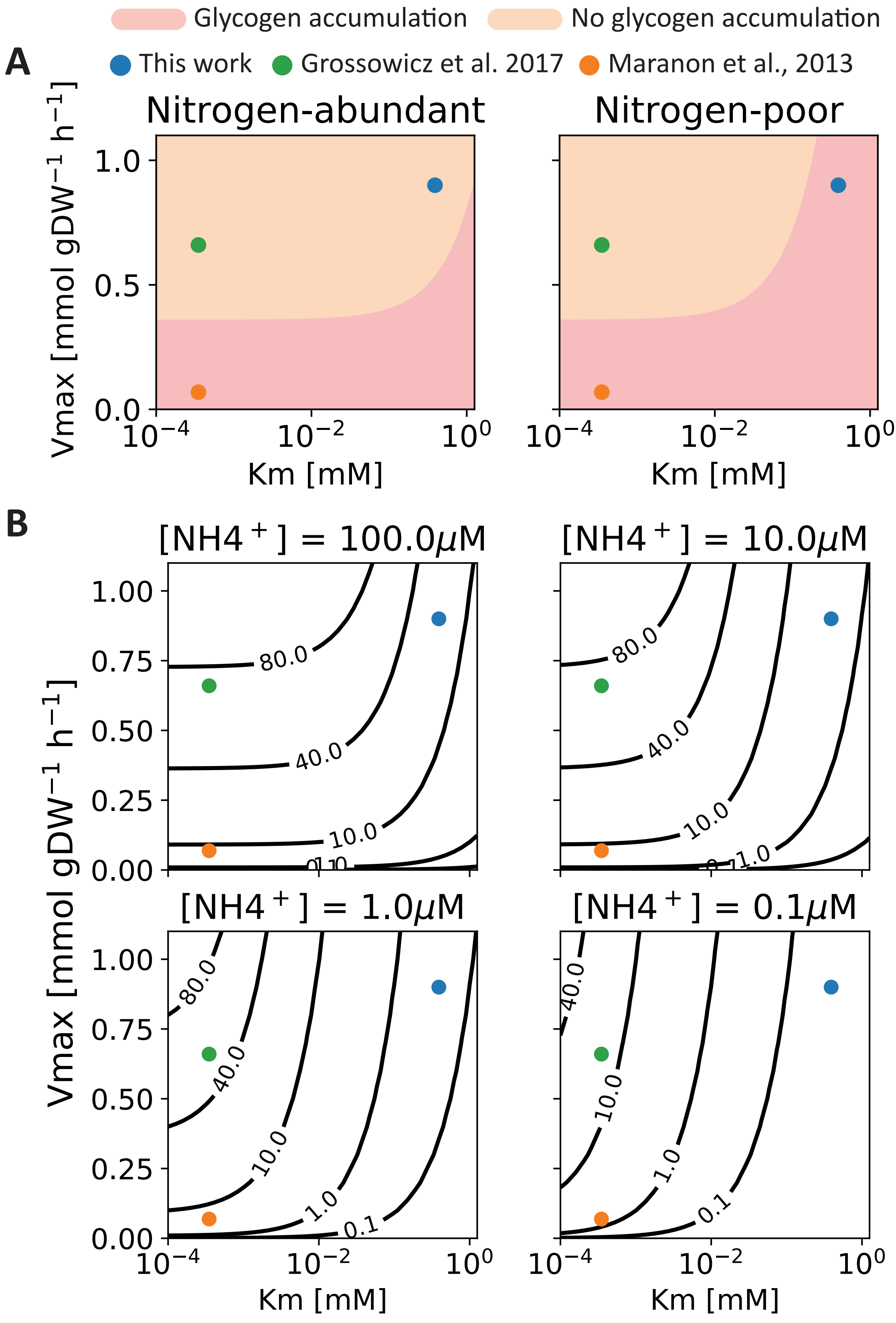

Glycogen accumulation
No glycogen accumulation
A
This work
Grossowicz et al. 2017
Maranon et al., 2013
Nitrogen-abundant
Nitrogen-poor
Vmax [mmol gDW^{-1} h^{-1}]
Km [mM]
B
[NH4$^+$] = 100.0μM
[NH4$^+$] = 10.0μM
[NH4$^+$] = 1.0μM
[NH4$^+$] = 0.1μM
80.0
40.0
10.0
1.0
0.1

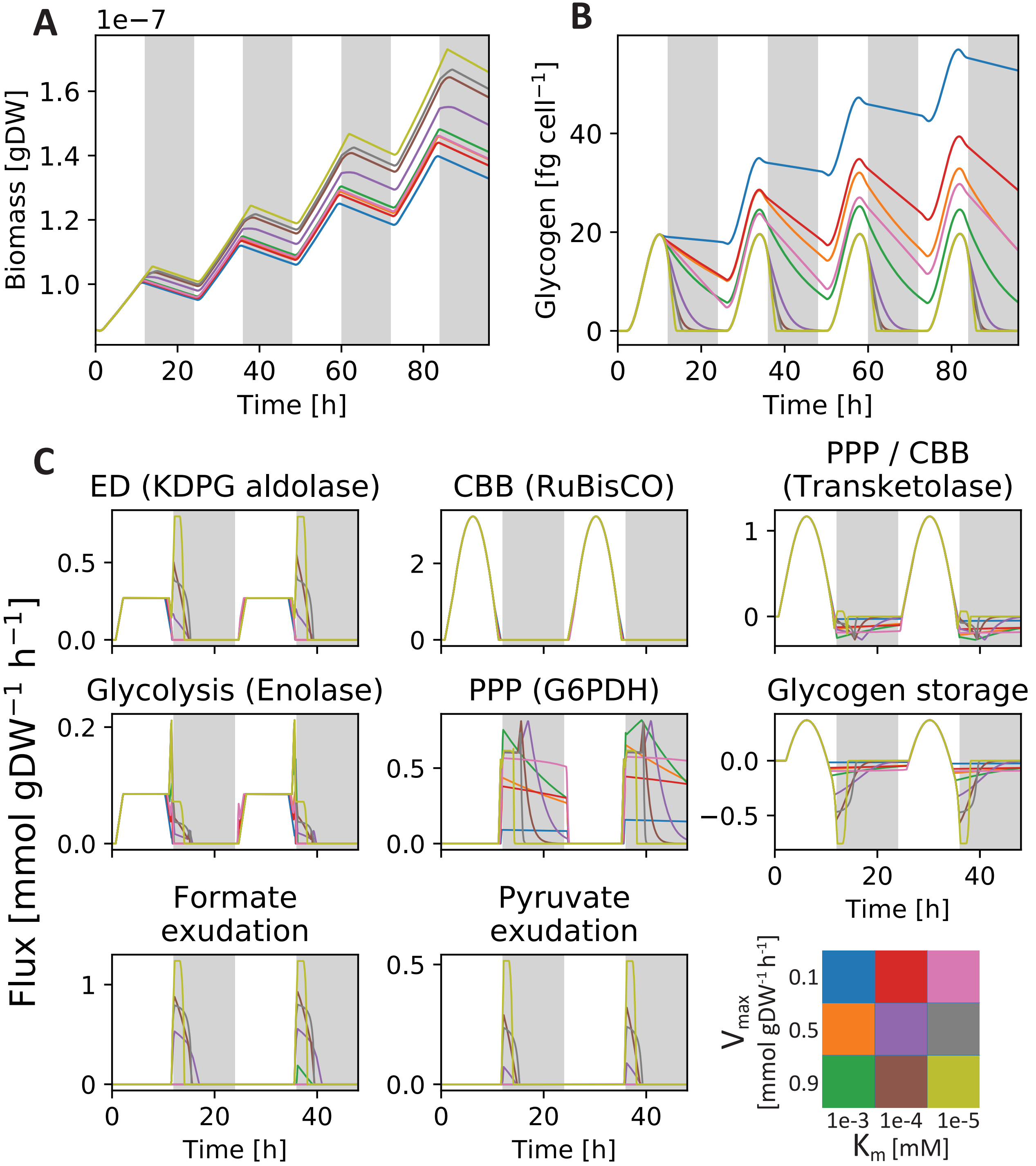

A
1e−7
Biomass [gDW]
1.0
1.2
1.4
1.6
Time [h]
B
Glycogen [fg cell−1]
0
20
40
C
Flux [mmol gDW−1 h−1]
ED (KDPG aldolase)
CBB (RuBisCO)
PPP / CBB (Transketolase)
Glycolysis (Enolase)
PPP (G6PDH)
Glycogen storage
Formate exudation
Pyruvate exudation
Vmax [mmol gDW-1 h-1]
0.1
0.5
0.9
1e-3
1e-4
1e-5
Km [mM]

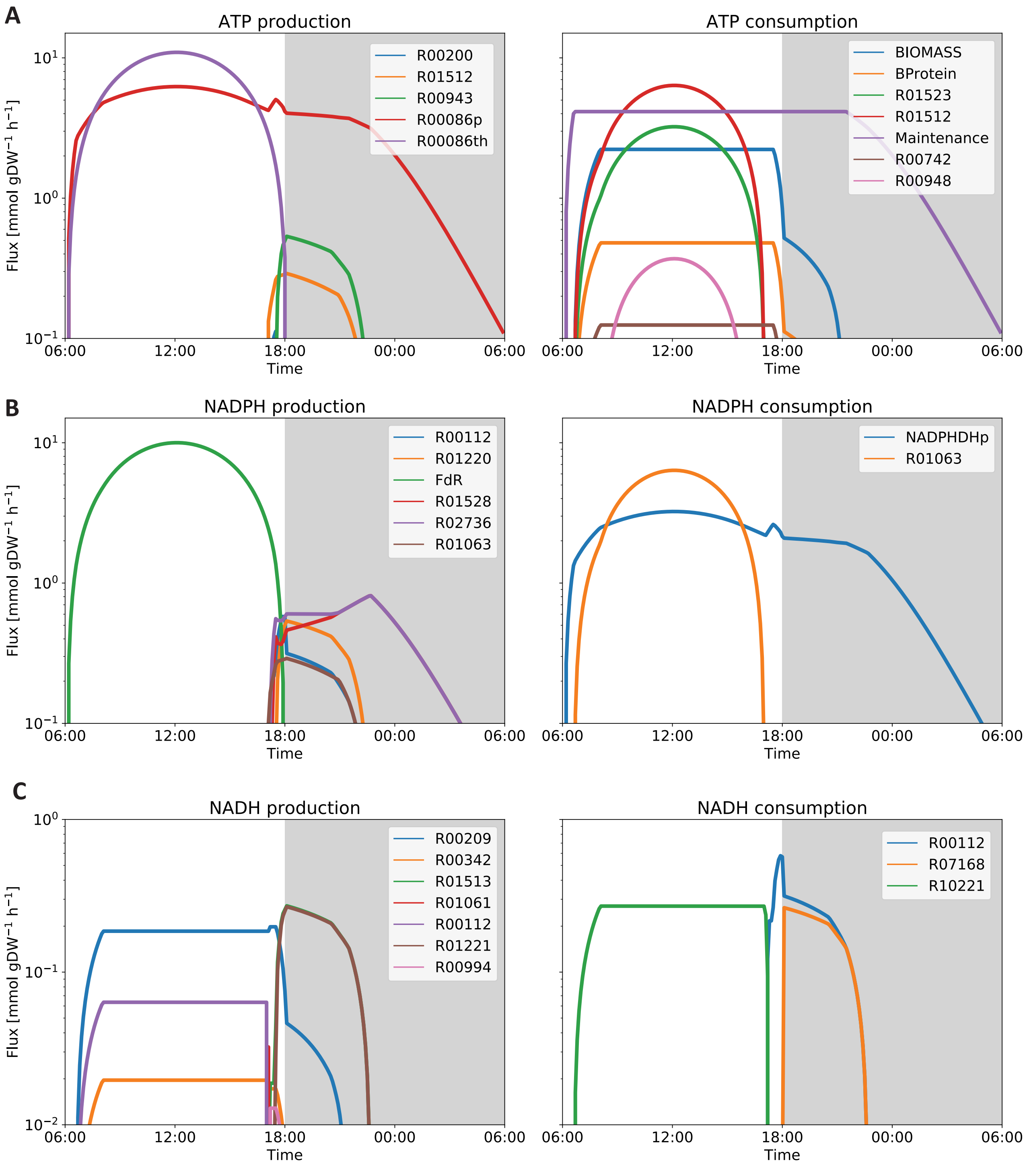
A
ATP production
R00200
R01512
R00943
R00086p
R00086th
ATP consumption
BIOMASS
BProtein
R01523
R01512
Maintenance
R00742
R00948
B
NADPH production
R00112
R01220
FdR
R01528
R02736
R01063
NADPH consumption
NADPHDHp
R01063
C
NADH production
R00209
R00342
R01513
R01061
R00112
R01221
R00994
NADH consumption
R00112
R07168
R10221
Flux [mmol gDW^{-1} h^{-1}]
Time
06:00
12:00
18:00
00:00
06:00

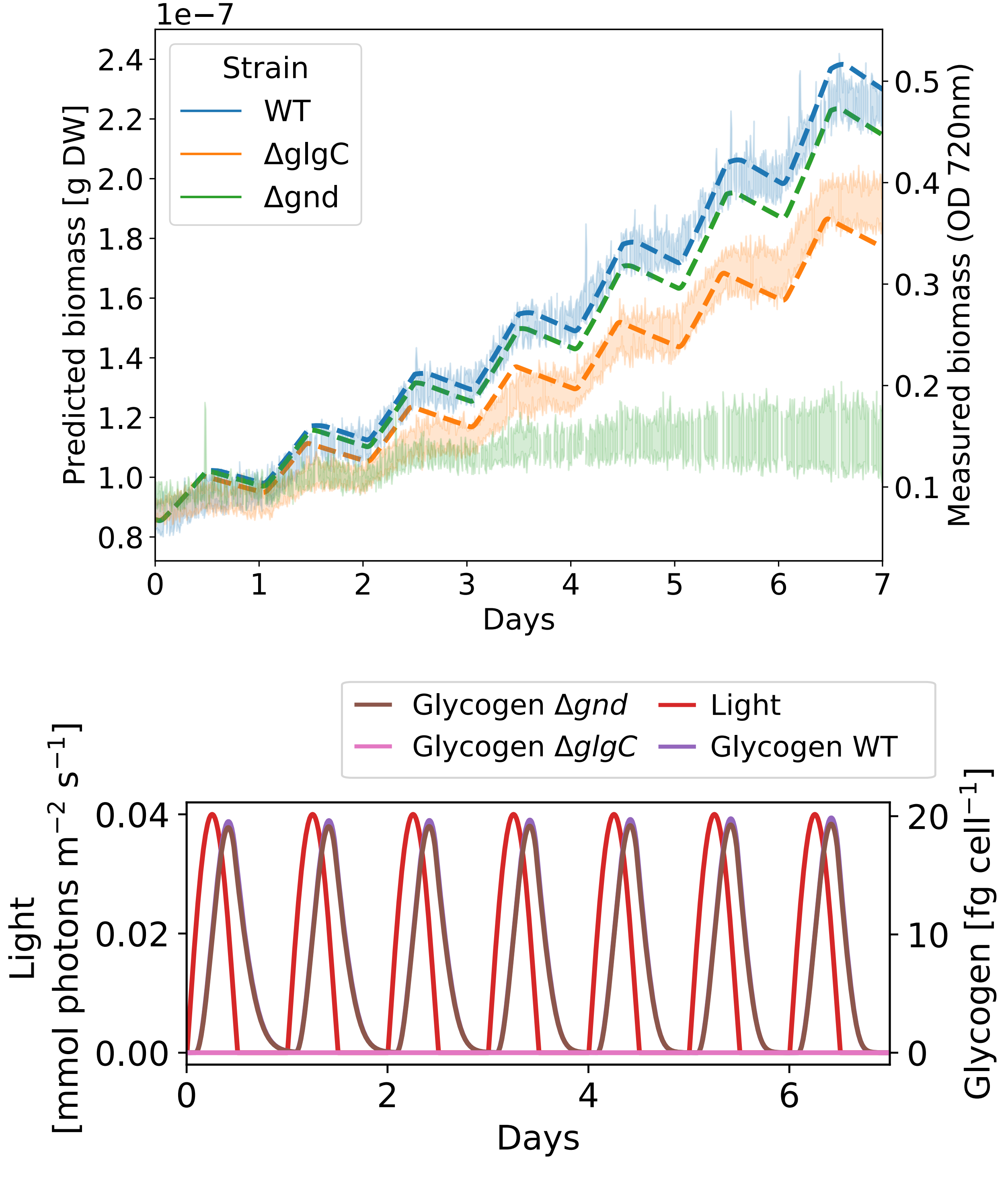

1e−7
Strain
WT
ΔglgC
Δgnd
Predicted biomass [g DW]
Measured biomass (OD 720nm)
Days
Glycogen Δgnd
Light
Glycogen ΔglgC
Glycogen WT
Light
[mmol photons m−2 s−1]
Glycogen [fg cell−1]
Days
