# Supplementary material for: Dynamic Allocation of Carbon Storage and Nutrient-Dependent Exudation in a Revised Genome-Scale Model of Prochlorococcus
Source: Front Genet. 2021 Feb 9;12:586293. doi: 10.3389/fgene.2021.586293 (PMC7900632; doi:10.3389/fgene.2021.586293)
Supplement: Supplementary Material 2 — Memote snapshot report of iSO595. [file Data_Sheet_2.pdf]

Independent Section

Contains tests that are independent of the class of modeled organism, a model's complexity or types of identifiers that are used to describe its components. Parameterization or initialization of the network is not required. See readme for more details.

Consistency

Stoichiometric Consistency	34.4%
Mass Balance	76.2%
Charge Balance	100.0%
Metabolite Connectivity	100.0%
Unbounded Flux In Default Medium	85.8%
Sub Total	66%

Annotation - Metabolites

Presence of Metabolite Annotation	0.0%
Metabolite Annotations Per Database	Info
pubchem.compound	0.0%
kegg.compound	0.0%
seed.compound	0.0%
inchikey	0.0%
inchi	0.0%
chebi	0.0%
hmdb	0.0%
reactome	0.0%
metanetx.chemical	0.0%
bigg.metabolite	0.0%
biocyc	0.0%
Metabolite Annotation Conformity Per Database	Info
pubchem.compound	0.0%
kegg.compound	0.0%
seed.compound	0.0%
inchikey	0.0%
inchi	0.0%
chebi	0.0%
hmdb	0.0%
reactome	0.0%
metanetx.chemical	0.0%
bigg.metabolite	0.0%
biocyc	0.0%
Uniform Metabolite Identifier Namespace	0.0%
Sub Total	0%

Annotation - Reactions

Presence of Reaction Annotation	0.0%
Reaction Annotations Per Database	Info
rhea	0.0%

Specific Section

Covers general statistics and specific aspects of a metabolic network that are not universally applicable. See readme for more details.

SBML

SBML Level and Version	SBML Level 3 Version 1
FBC enabled	true

Basic Information

Model Identifier	COBRA Model
Total Metabolites	802
Total Reactions	994
Total Genes	595
Total Compartments	5
Metabolic Coverage	1.67

Metabolite Information

Unique Metabolites	802
Duplicate Metabolites in Identical Compartments	0
Metabolites without Charge	0
Metabolites without Formula	0
Medium Components	24

Reaction Information

Purely Metabolic Reactions	790
Purely Metabolic Reactions with Constraints	5
Transport Reactions	101
Transport Reactions with Constraints	2
Thermodynamic Reversibility of Purely Metabolic Reactions	1.00
Reactions With Partially Identical Annotations	0.00
Duplicate Reactions	0.00
Reactions With Identical Genes	0.44

Gene-Protein-Reaction (GPR) Associations

Reactions without GPR	164
Fraction of Transport Reactions without GPR	0.76
Enzyme Complexes	190

Biomass

Biomass Reactions Identified	2
Biomass Consistency	Info
BIOMASS	0.00
BiomassTRANS	0.00
Biomass Production In Default Medium	Info
BIOMASS	0.10

metanetx.reaction	0.0%
bigg.reaction	0.0%
reactome	0.0%
ec-code	0.0%
brenda	0.0%
biocyc	0.0%
Reaction Annotation Conformity Per Database	Info
rhea	0.0%
kegg.reaction	0.0%
seed.reaction	0.0%
metanetx.reaction	0.0%
bigg.reaction	0.0%
reactome	0.0%
ec-code	0.0%
brenda	0.0%
biocyc	0.0%
Uniform Reaction Identifier Namespace	100.0%
Sub Total	25%

Annotation - Genes

Presence of Gene Annotation	0.0%
Gene Annotations Per Database	Info
refseq	0.0%
uniprot	0.0%
ecogene	0.0%
kegg.genes	0.0%
ncbigi	0.0%
ncbigene	0.0%
ncbiprotein	0.0%
ccds	0.0%
hprd	0.0%
asap	0.0%
Gene Annotation Conformity Per Database	Info
refseq	0.0%
uniprot	0.0%
ecogene	0.0%
kegg.genes	0.0%
ncbigi	0.0%
ncbigene	0.0%
ncbiprotein	0.0%
ccds	0.0%
hprd	0.0%
asap	0.0%
Sub Total	0%

Annotation - SBO Terms

BIOMASS	false
BiomassTRANS	false
Biomass Production In Complete Medium	Info
BIOMASS	54.20
BiomassTRANS	54.20
Blocked Biomass Precursors In Default Medium	Info
BIOMASS	0
BiomassTRANS	0
Blocked Biomass Precursors In Complete Medium	Info
BIOMASS	0
BiomassTRANS	0
Ratio of Direct Metabolites in Biomass Reaction	Info
BIOMASS	0.00
BiomassTRANS	0.00
Number of Missing Essential Biomass Precursors	Info
BIOMASS	37
BiomassTRANS	37

Energy Metabolism

Non-Growth Associated Maintenance Reaction	Errored
Growth-associated Maintenance in Biomass Reaction	Info
BIOMASS	false
BiomassTRANS	false
Number of Reversible Oxygen-Containing Reactions	8
Erroneous Energy-generating Cycles	Info
MNXM3	Skipped
MNXM63	Skipped
MNXM51	Skipped
MNXM121	Skipped
MNXM423	Skipped
MNXM6	Skipped
MNXM10	Skipped
MNXM38	Skipped
MNXM208	Skipped
MNXM191	Skipped
MNXM223	Skipped
MNXM7517	Skipped
MNXM12233	Skipped
MNXM558	Skipped
MNXM21	Skipped
MNXM89557	Skipped

Network Topology

Universally Blocked Reactions	79
Orphan Metabolites	9

Reaction General SBO Presence	0.0%	
Metabolic Reaction SBO:0000176 Presence	0.0%	
Transport Reaction SBO:0000185 Presence	0.0%	
Exchange Reaction SBO:0000627 Presence	0.0%	
Demand Reaction SBO:0000628 Presence	Skipped	
Sink Reactions SBO:0000632 Presence	Skipped	
Gene General SBO Presence	0.0%	
Gene SBO:0000243 Presence	0.0%	
Biomass Reactions SBO:0000629 Presence	0.0%	
Sub Total	0%	X2
Total Score	28%	

Total Score

28%

Score per Category

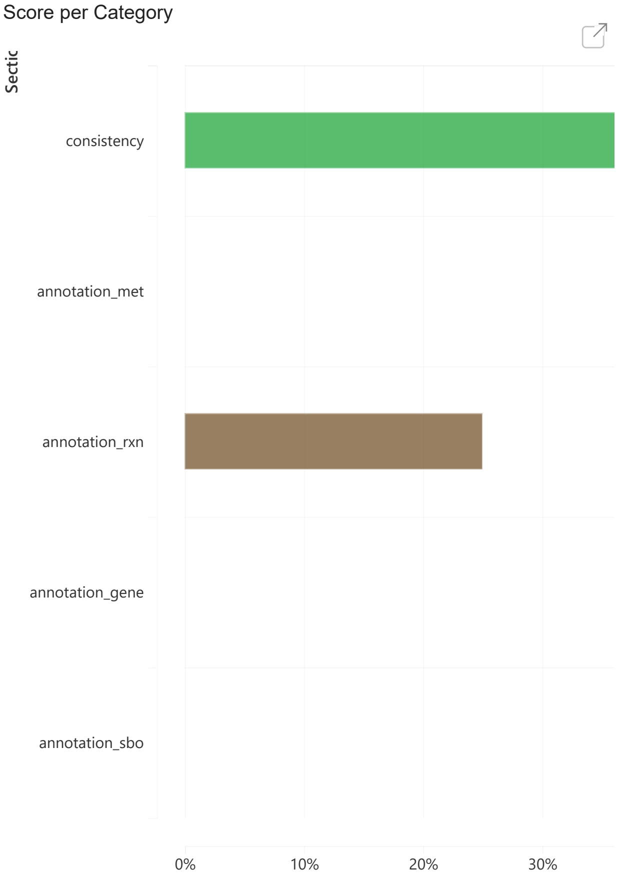

Metabolite Production In Complete Medium	101
Metabolite Consumption In Complete Medium	103

Matrix Conditioning

Ratio Min/Max Non-Zero Coefficients	0.00
Independent Conservation Relations	35
Rank	767
Degrees Of Freedom	227

Experimental Data Comparison

Growth Prediction	Skipped
Gene Essentiality Prediction	Skipped

Misc. Tests

Environment

Python Version	3.7.3
Platform	Windows
Memote Version	0.9.13
